# Dynamic connectedness of green bond with financial markets of European countries under OECD economies

**DOI:** 10.1007/s10644-022-09430-3

**Published:** 2022-08-16

**Authors:** Miklesh Yadav, Nandita Mishra, Shruti Ashok

**Affiliations:** 1 ACCF, Amity University, Noida, India; 2 Department of Management and Engineering, Linköping University, Linköping, Sweden; 3 Bennett University, Greater Noida, India

**Keywords:** Co-movement, Green bonds, OECD countries, Stock market, COVID-19, G10, G11, G19, Q01

## Abstract

This paper examines the dynamic connectedness between green bonds and OECD financial markets of European countries. The study is conducted on daily price of green bonds and selected European stock markets from January 27, 2015, to August 4, 2021. Top ten European countries namely Luxembourg, Switzerland, Norway, Denmark, Germany, Netherlands, Iceland, Austria, Sweden, and Belgium are included within the OECD economies. The study uses Diebold and Yilmaz and Barunik & Krehlic tests to examine the connectedness between the economies and green bonds in short, medium, and long term. Result exhibits volatility across all frequency cycles. Brussel Stock Exchange and Euronext Amsterdam are identified as high-risk markets in the OECD European market. Evidence emerging from this study advocate the inclusion of green bonds in these financial markets for shorter time periods only. Results from this study are expected to have practical implications for portfolio managers, investors, and market regulators, suggesting incorporation of green bonds in investor portfolio for efficient diversification of risk.

## Introduction

Green bonds are fast evolving as a new financing tool, gaining significant traction in the context of climate change risk. Climate change—an alarming global phenomenon, has inflicted considerable damage to environment worldwide. With the global threat due to climate change looming large, last few years have witnessed numerous initiatives undertaken to promote environmental awareness. Among them, promoting the usage of renewable energy to reduce carbon footprints and discourage fossil fuel consumption is significant (Taghizadeh-Hesary and Yoshino, 2020). The primary objective of Paris Agreement was to redirect funds to projects, that foster climate resilience, while reducing carbon emissions. With the transition to reduced reliance on carbon as a fuel, economies now require huge investments to fund environment friendly projects (Tiron-Tudor et al. 2021). To achieve the Paris Agreement climate targets and the UN Sustainable Development Goals (SDGs), enormous commitment of financial resources in climate friendly projects and investment opportunities is imperative (Reboredo and Ugolini, 2020). The many ways through which finance sector acts as a catalyst for advancing sustainability include promoting investments into sustainable projects through innovative financial products. Since green bonds facilitate attainment of the objectives of Paris Agreement, investments in them have become a necessity. International Capital Market Association (2018) defines green bonds as “any bond instrument where the proceeds will be exclusively applied to finance or refinance, in part or full, to new or existing eligible green projects.” The main difference between conventional and green bonds lies in the end use of proceeds; green bonds employ all the proceeds in projects that are focused on renewable energy, green building, clean infrastructure and energy efficiency (Flammer, 2020; Nguyen et al. 2020). Green bonds are also called sustainable bonds, as they promote sustainable development and have emerged as a plausible source of sustainable financing for institutional investors (pension funds, insurance companies, mutual funds, and sovereign wealth funds), allowing them to divert funds to sustainable infrastructure investments.

Among the latest financial innovations, variety of instruments like blue bonds and brown bonds are also gaining prominence. Aimed at fulfilling sustainable development goals (SDG) of clean water and life below water, blue bonds (subset of green bonds) are a relatively newer form of sustainable bonds, financing ocean conservation projects. Brown bonds enable brown industries (companies that transmit significant emissions) to raise funds, facilitating transition to improved sustainable models. Even with the many options available in sustainable bonds, green bonds are more popular and much in demand than other similar sustainable bonds. Green bonds are certified as per the standards developed by the Climate Bonds Initiative, whereas no such framework exists for brown or blue bonds. Application of proceeds from blue bonds and brown bonds is limited, whereas green bonds offer a wider spectrum of proceeds utilization in environment related projects.

The green bond market constitutes 1.5% of the total bond market with cumulative issuances at $1.79 trillion till date (King et al. 2021). According to CBI (2022), global green bonds market has reached over 1.795 trillion USD, with investments worth $145.6 billion till date in the year 2022 alone. The first green financing was done in 2007 by European Investment Bank followed by World bank investment in 2008. The European Green deal was a significant step towards reinforcement of sustainable financing. With EU’s aim to become a carbon-neutral economy by 2050, this deal intended to transform EU into a fair, sustainable, and prosperous society. With considerable focus on promoting green bonds, EU has time and again launched many initiatives. Notable among them were ‘EU Green Bonds Standards’ that aimed to enhance the transparency and standardization of green markets and promote green bonds issuance to boost investors’ confidence. Another initiative was the adoption of EU taxonomy identifying environmentally sustainable activities to smoothen the implementation of EU Green deal (Technical Expert Group 2019). With the confusion around the definition and meaning of sustainable investments, adoption of EU taxonomy was intended to provide well-established and recognized definition to the investors, companies, governments, and policymakers. It was expected that such a move would reduce greenwashing while encouraging circular economy and other climate related initiatives.

**Fig. 1:**
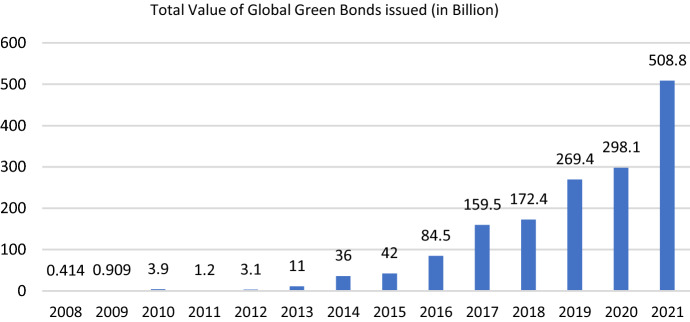
Global Green Bond Issuance (2008–2021). Source: https://www.climatebonds.net/market/data/#country-map accessed on 31st May, 2022

EU has always led green bonds issuance, constituting 48% of total global issuances in 2020 (Spinaci, S. 2022). In terms of volume, Sweden, Spain and Germany were among the top 10 countries to issue green bonds. In the year 2021, annual green bond issuance crossed $510 billion (see Fig. 1), Europe being the highest contributor by issuing green bonds worth $265 billion. EU, over the years, has advocated the adoption of green bonds and has promoted sustainability by introducing new laws and regulations. Considering EU’s initiatives around sustainability, carbon neutrality and green financing, this study examines the top ten EU economies and examines the connectedness of green bonds with the financial markets in selected EU economies. With limited studies establishing the connection between green bonds and attainment of Sustainable Development Goals (SDGs) (Le, and Taghizadeh-Hesary, 2020; Taghizadeh-Hesary and Yoshino, 2019), this study aims to establish green bonds as one of the efficient ways to achieve SDGs by 2030.

Due to the many advantages of green bonds, they constitute a significant portion of active trades in markets world over. Extensive research is being carried on green bonds, ranging from analysis of their general characteristics to exploring the association of green bonds with the equity market. The mechanism of risk transmission between equity and bond markets plays a vital role in risk management and portfolio composition. Compared to equity, bonds as an asset class are relatively stable, potentially offsetting the risk of stocks in a portfolio. Green bonds have emerged as an environment friendly instrument in the fixed income product category and are increasingly included in investors’ portfolios, for improved risk divergence and mitigation. Inclusion of Green bonds is asset management that has exhibited positive impact on equity performance, improved liquidity, and increased short-run firm value (Flammer, 2020). Much of the existing literature, therefore, has explored the dynamic relationship between the equity and bond markets (Cappiello et al., 2006; Christiansen, 2010; Chuliá and Torro, 2008). Owing to the inherent characteristics of green bonds as a stable form of investment option, analyzing the connectedness of green bond market with and stock markets becomes imminent. Such research is expected to assist investors in improved organization and management of their portfolios. However, since green bonds are relatively newer investment option, existing academic research on this topic is largely insufficient. With EU leading the pack in green bond issuance, it becomes imperative to explore the connectedness of green bond market with the stock market in European countries. With limited studies exploring the relationship in European region, this study attempts to fill in this gap by analyzing the dynamic connectedness between top ten European countries (OECD) and the green bond financial markets.

Results of this study are expected to have meaningful implications both on theoretical and practical grounds in the following ways: Firstly, due to limited studies on connectedness between OECD financial markets and green bonds, this study will contribute to the existing literature by evidencing this relationship. Considering the recent developments and buzz around green bonds in European countries, it becomes crucial to understand and evaluate this connectedness. Secondly, application of both Diebold-Yilmaz (2012) and Barunik & Krehlic (2017) tests lends credence to this study. Connectedness, as suggested by Diebold and Yilmaz, would analyse the association of variables, while evaluating the share of forecast error variation in different locations due to shocks. Barunik and Krehlik test would extend this connectedness to the framework of frequency domain. Since this paper effectively employs both these tests, it is expected that the results would have meaningful implications for policymakers, investors, and academicians. Thirdly, the findings of this study can guide various investor classes like hedge funds, intra-day traders (short-term horizon), pension funds, insurance companies and sovereign wealth funds (medium- to long-term) to identify suitable investment opportunities. Finally, since, inclination for investment in safer options assumes prominence during pandemic times like COVID-19, results from this study can promote investments in countries that encourage mobilization of financial resources in green bonds to support a climate-resilient economy.

The rest of the paper is divided into different sections. Next section expounds on the review of existing literature followed by methodology and Result in Sect. 3 and 4, respectively. Section 5 is of conclusion and the policy implication of the study.

## Literature review

Green bond has emerged as a promising investment option (Banga, 2019), with its issuance increasing by almost five times in the last five years and is further expected to reach $1 trillion per year by 2030 (Fatin, 2019). Recent developments have moved the attention from economic or financial indicators towards sustainability. Therefore, green bonds have become a much-debated topic in the literature.

International bodies and governments are becoming increasingly mindful of contribution by green bonds in building a robust economy. It is expected that going forward, many countries would include green bonds in their portfolios to tackle climate issues. Increasing awareness and popularity of green bonds has attracted plentiful academic interest. But due to the heterogeneity between the sample, studies are more focused towards the qualitative studies and not on quantitative ones (Zhou and Cui, 2019). Many researchers have established that green bonds can be helpful in combating climate change (Flaherty et al. 2017). On the other hand, few studies have also proved that green bond leads to additional cost of certification and also limits the type of project (Tolliver et al. 2020). Tolliver et al. (2020) not only highlighted obstacles in development of green bonds but also provided with possible solution to these problems. One of the solutions provided in the study was that green bond should be issued by the national and local government. Multilateral development banks should also be involved in the green bond issuance and investment. Many government agencies, local and international bodies have started channelizing their budget to support projects which deal with climate issues (Zhou & Cui, 2019). Studies have investigated various countries to determine whether governments have been able to derive the expected benefits by investing in green bonds.

Another strand of researcher has focused on the pricing of the green bonds and has compared green bonds with the conventional bonds. Löffler et al. (2021) in their study focused on the difference between the yield of conventional bonds and the green bonds. They argued that the yield of conventional bonds is higher than the green bonds. But few researchers have reported exactly opposite trend where green bonds have shown better yield (Lautsi, 2019). Agliardi & Agliardi (2019) highlighted that the presence of “green premium” makes green bond cheaper than other bonds. MacAskill et al. (2020) in their study emphasize that green premium is because of different type of bonds, bond rating, third party assessment and type of issuer. Few studies show that green bond has positive impact on the firm value, liquidity, and stock price of company (Flammer, 2020; Kuchin et al. 2019; Tang and Zhang, 2020). These mixed results can be because of different sample sizes, time, or methodology but eventually the studies don’t agree on any common grounds.

Few researchers have also tried to emphasize on the methods which may be adopted to lower the cost of green bonds. Agliardi and Agliardi (2019) suggests that if governments lower the tax, improve the credit quality and green awareness, the cost of capital of green bonds will be reduced which will boost the issuance of green bonds. Li et al. (2020) also did similar study with China as base country and found that corporate social responsibility, credit rating and certification of green will lower the cost of issuance of green bonds. Bachelet et al. (2019) also emphasized on third party verification to improve the yield of the green bonds. A latest study by Russo et al. (2021) has proven that the performance of green bond depends on the financed project and alignment of the issuer. Institutional issuers have advantage over the private issuers. Interestingly, there is no consensus on the risk and premium of green bonds. Scholars have used co-movement for studying the impact of financial crisis. Uddin et al. (2013) examined the co-movement between Germany and other important International Stock market and found that the difference in the co-movement dynamics could be the result of the financial crisis. Tiwari et al. (2015) studied the crisis of Europe and USA and their work suggested that frequency and time varies co-movement of the uncertainty indices. A recent study by Zhao et al. (2021) measures renewable energy firm-level pure innovation efficiency, green productivity, technical efficiency, scale efficiency and total investment efficiency from micro input–output factors.

One more very important aspect of green bonds is the determinants of green bonds. Studies related to determinants identify issuer traits, security characteristics, financial health of issuer, rating and market features as some of the important determinants of green bond issue (Chiesa and Barua, 2019). Tolliver et al. (2020) studied the green bonds of 49 countries and found that macro-economic, national level contributions and institutional factors impact the issue of green bonds. They connected Paris agreement objectives with the issue of green bonds. Anh Tu et al. (2020) did a similar study on developing economies and found that the factors impacting issue of green bonds were mainly legal structure, rate of interest and the stability of the economy. In China, where we see a great amount of issue of green bonds, positive impact on stock price, corporate social responsibility and profitability were some of the important determinants of issuance of green bonds (Zhou and Cui, 2019).

One more very important aspect of green bond is the impact of green bond issuance on the market reaction and implications of economic value. Flammer (2021) found that the stock market reacts in a positivity way to the announcement of the green bond and this reaction is even more strong in case of certified bonds. In their study, Pham and Huynh (2020) measured the investor’s attention in terms of ‘Google Search Volume Index’ and found that it was positive for green bonds. At the country level, few studies have tried to find the determinants of development of government bond market and found that economy size, corruption level, distance from equator, openness towards trade, level of bureaucracy and banking system influence the bond market (Claessens et al. 2007; Eichengreen and Luengnaruemitchai 2004). Few studies highlighted that climate awareness among the investors and attitude of government and policymakers are important determinants of green bond issue (Banga, 2019). Scholars across the world have conducted many studies on linking energy commodities with economic development. This provides an interesting dimension, relating energy commodities with green bonds. Analysis of literature reveals sufficient research conducted on energy commodities and the linkage with sustainable development (Bruno & Sachs, 1982; Gisser & Goodwin, 1986). Existing studies also explore the impact of energy commodities volatility (such as oil) on stock market performance (Dutta et al. 2017; Noor & Dutta, 2017). The studies confirm the presence of volatility transmission from oil markets to equity markets and possible portfolio diversification strategies amidst the oil price risk. However, studies examining the relationship between green bonds and energy commodity are limited. Few studies have associated energy commodities with utility equities (Boyer & Filion, 2007).

As one of the most devastating global events, COVID-19 posed serious challenges to the over all economy. Although the COVID-19 pandemic has damaged the global economy, it has also led to an expansion in the allocation of green and renewable energy (Wan et al. 2021). This has led to increase in the number of green bonds (Chai et al. 2022). Yi et al. (2021) also confirmed it through their research that COVID-19 has improved the green bond market. Furthermore, recent studies have underlined the connectedness between green bonds, clean energy and stock price. Kung et al. (2022) in their study established a positive impact of green bonds on the bio-energy. While existing research has evidenced impact of unidirectional stock market spill over on green bond market (Gao et al. 2021), the need for further research to confirm COVID-19 impact on green bond markets is absolutely essential.

Overall, a great deal of variation and discrepancy can be found in the literature. We found that many studies have been conducted on China but results from China cannot be implemented on EU countries because of difference in the definition of the green financing. In China, coal finance is considered as green, whereas in Europe, it is not considered as green (Gilchrist et al. 2021). This creates a huge gap in the literature. Review of existing literature reveals that despite existing research conducted on green bonds, studies lack consensus. Limited evidence of relationship between green bonds and financial markets exploring their role in investment portfolios is available. The EU countries are leading in taking sustainability initiatives and also in terms of volume and frequency of issues green bonds (Cheong and Choi, 2020; Halkos et al. 2020). Chiesa and Barua, 2019 also argues that there is difference in impact factors in emerging and non-emerging economies. Relating green bonds to the economy of EU counties has not been studied in detail and the impact of COVID-19 widens the gap in the literature. This study attempts to fill this gap by studying the connectedness of green bonds with the financial markets of top 10 EU countries under OECD economies. The green bond market is demonstrating different level of development around the world. There are still many countries where green bonds are still not issued. Our study sheds light on the EU countries because of their exceptional initiatives towards sustainability. The result of this paper is likely to be important for industry, investors, government, and academicians.

## Data and econometric models

### Data

The study examines the dynamic connectedness of green bond with financial markets of European countries under OECD economies. The proxies of green bond are Barclays MSCI Green Bond Index (BMSCIGB) and S&P Dow Jones Green Bond Index (SPDR). Generally, green bond is issued for the financing of projects which focuses on environment/climate (Saeed et al. 2020). For OECD economies, top ten European countries like Luxembourg, Switzerland, Norway, Denmark, Germany, Netherlands, Iceland, Austria, Sweden, and Belgium are taken into consideration. The proxies of these countries stock markets are Luxembourg stock exchange (LSE), Six Swiss exchange (SSE), Oslo stock exchange, Denmark stock market index (DSME), Frankfurt stock exchange (FFSE), Euronext Amsterdam (ENA), Nasdaq Iceland (NSDI), Austria stock exchange (ASE), Nasdaq Stockholm AB (NSDS) and Brussels stock exchange (BSE). The purpose behind considering these markets is that green bonds issued in EU economies account for 40% of global green bonds issuance (Dan et al. 2021). EU, over the years, has advocated the adoption of green bonds and has promoted sustainability by introducing new laws and regulations. Considering EU’s initiatives around sustainability, carbon neutrality and green financing, this study examines the top ten EU economies and examines the connectedness of green bonds with the financial markets in selected EU economies. The adjusted daily price of green bond and European stock markets using Bloomberg from January 27, 2015, to August 4, 2021, are studied. Further, raw series (adjusted closing price) of constituent series is converted into log return making logarithmic differences of two successive days prices (Yadav & Pandey, 2020). The following formula has been used to convert into log return series:

$$R_{i,t} = {\text{log}}\left( {\frac{{P_{i,t} }}{{\left( {P_{i,t - 1} } \right)}}} \right)$$

where *R*_*i, t*_ represents logarithmic return at time t, while *P*_*i,t–1*_ and *P*_*i,t*_ are the daily closing prices of i*th* fund on successive days. Table 1 provides the detailed data description of the green bond and European financial markets:

**Table 1:** Description of European financial market and green bond

Market	Asset	Acronyms	Source
European financial markets	Luxembourg stock exchange	RLSE	Bloomberg
	Six Swiss exchange	RSSE	
	Oslo stock exchange	ROSE	
	Denmark stock market index	RDSME	
	Frankfurt stock exchange	RFFSE	
	Euronext Amsterdam	RENA	
	Nasdaq Iceland	RNSDI	
	Austria stock exchange	RASE	
	Nasdaq Stockholm	RNSDS	
	Brussels stock exchange	RBSE	
Green bond	Barclays MSCI green bond index	RBMSCIGB	
	S&P Dow Jones green bond index	RSPDR	

Author’s own presentation

### Econometric models

To examine the dynamic connectedness, the study employs Diebold and Yilmaz (2012) and Barunik and Krehlik (2017) models. Both are used to check for frequency connectedness among variables. The details of these two models are explained as below:

#### Diebold and Yilmaz (2012)

Diebold and Yilmaz (2012) model are used to check for frequency connectedness among the variables. This study employs Diebold and Yilmaz (2012) model to quantify the level of connectedness among the variables by measuring the breakdown of forecast error variance. By focussing on the variance composition, this study identifies both directional and net spill overs between the various markets and the green bonds. Diebold and Yilmaz (2012) model are primarily used as a measuring tool and rather than as an application to identify statistical significance. The model disintegrates the forecast error variance of variable *i* into portions and attributes to the many variables present. The K-variable VAR(*p*) system can be defined as follows:

1 $$y_{t} = c + A_{{1}} y_{t - 1} + A_{{2}} y_{{t - {2}}} + \cdots + A_{p} y_{t - p} + u_{t} ,$$

where a *y*_*t*_ denotes the *K* × 1 vector of the variables at time *t*, *c* denotes the *K* × 1 vector of the constants, and *A* denotes the coefficients’ *K* × *K* dimension matrix. Equation (1) can be rewritten in a simpler form as follows:

2 $$Y_{t} = C + AY_{t - 1} + U_{t} ,$$

In case of multivariate time series, the model initially fits a vector autoregressive model and then creates a H period forecast. Estimating the VAR model, we employ a variance decomposition to examine how much each.

variable contributes to explaining other variables. Further, forecast error variance is segregated basis the shocks to each variable for due to the other variables at time *t*. *d*^*H*^ denotes the *ij*-th H-step forecast error variance, i.e. *d*^*H*^ represents the *ij* fraction of variable *i*’s H-step forecast error variance due to shocks in variable *j*. It is to be noted that *d*^*H*^*, i*, *j* = 1, *· · · N*, *i ƒ* = *j*. Cholesky’s decomposition of the variance covariance matrix Ω_*µ*_ = *E*(*µ*_*t*_*µ*_*t*_^′^) is employed to estimate the lower triangular matrix *P*. Moreover, Φ_*j*_ = *JA*^*j*^* J*^′^, where *J* = [*I*_*K*_, 0,…, 0]. The contribution of variable *k* to variable *i* is then given by the following equation:

$$\theta _{{ik,H}} = {\mkern 1mu} \sum H - 1e_{i}^{\prime } \Theta _{j} e_{k} \;\;2/{\text{MSE}}\left[ {y_{{i,t}} \left( H \right)} \right].$$

Following Diebold and Yilmaz, we measure the connectedness of the system’s variables to summarize all elements in *θ*(*H*) from 1 to *K*. Connectedness is measured by

3 $$C_{H} = \frac{1}{K}\sum\limits_{{ij = 1}}^{{j = 0}} {K_{{ij}} {\mkern 1mu} \theta ^{H} (i/ = j),}$$

which excludes all diagonal elements from the system to ensure that the total connectedness ranges from 0 to 1. Therefore, this measure examines the degree to which each system component’s contribution to variations is caused by something other than itself. All the system components are independent and without spillover effects when their value equals zero. In contrast, the system components are perfectly connected when this value equals one.

As the order of variables in the VAR system may affect the impulse response or variance decomposition results, we follow Diebold and Yilmaz’s work—in using the generalized variance decomposition approach developed by Koop et al. (1996) and Pesaran and Shin (1998)—to make our results more robust:

$$\theta _{{iK,H}}^{g} = \sigma _{{ii}}^{{ - 1}} \sum\nolimits_{{j = 0}}^{{H - 1}} {\left( {e_{i}^{\prime } \Phi _{j} \sum\nolimits_{u} {e_{k} } } \right)^{2} } /{\text{MSE}}\left[ {y_{{i,t}} \left( H \right)} \right].$$

### Barunik and Krehlik (2017)

After application of Diebold and Yilmaz test, this study employs Barunik and Krehlik (2017) method to explore the frequency dynamics of spill over and study the variance decomposition both for short-term, medium-term and long-term. In case connectedness is evident at higher frequencies, it suggests instant information processing that results in movement in one asset having an impact on the other asset, primarily in the short term. Connectedness found at lower frequencies indicates continuing shocks transmitted for longer periods term. Next, following Barunik and Krehlik [33], we discuss the frequency dynamics of the connectedness and describe the spectral formulation of variance decomposing; hence, we consider a frequency response function, Ψ e − iω = ∑ e − iωhΨh, which we can obtain as a Fourier transform of the coefficients.

$\Psi$, with $i = \sqrt { - 1}$. The generalized causation spectrum over frequencies ω (-π, π) is

$$\left( {f\left( \omega \right)} \right)_{{j,k}} \equiv \frac{{\sigma _{{kk}}^{{ - 1}} \left| {\left( {\left( {\Psi \left( {e^{{ - i\omega }} } \right)\sum } \right)_{{j,k}} } \right.} \right|^{2} }}{{\left( {\Psi \left( {e^{{ - i\omega }} } \right)\sum {\Psi ^{\prime } } \left( {e^{{ + i\omega }} } \right)} \right)_{{j,j}} }},$$

where Ψ e − iω = ∑h e − iωhΨh is the Fourier transform of the impulse response function Ψ and ( f (ω))j,k denotes the portion of the spectrum of the j-th variable under frequency ω due to shocks in the kth variable. As the denominator holds the spectrum of the j-th variable under frequency ω, we can interpret in the above equation as the quantity within the frequency causation. To obtain the generalized decomposition of variance decompositions under frequency ω, we weight the function (f (ω))j,k by the frequency share of the variance of the j-th variable. We can define the weighting function as in

$$\Gamma_{j} \left( w \right) = \frac{{\left( {\Psi \left( {e^{ - iw} } \right)\sum \Psi^{\prime } \left( {e^{ + iw} } \right)} \right)_{j,j} }}{{\frac{1}{2\pi }\int_{ - \pi }^{\pi } {\left( {\Psi \left( {e^{ - i\lambda } } \right)\sum \Psi^{\prime } \left( {e^{ + i\lambda } } \right)} \right)_{j,j} d\lambda } }}.$$

The above equation shows the power of the *j*-th ums of the frequencies to a constant value of 2*π*. We should note that although the Fourier transform of the impulse response is a complex number value, the generalized factor spectrum is the squared coefficients of the weighted complex numbers, and hence a real number. Formally, we begin to set up frequency band *d* = (*a*, *b*): *a*, *b* ∈ (− *π*, *π*), *a* < *b*.

$$\left( {\Theta_{d} } \right)_{j,k} = \frac{1}{2\pi }\int_{d}^{\infty } {\Gamma _{{\text{j}}} \left( w \right)\left( {f\left( w \right)} \right)}_{j,k} d\omega .$$

The generalized variance decomposition under the frequency band is relatively easy to formulate the connectedness measures under the frequency band using the spectral formulation of the generalized variance decomposition. We formulate the scaled generalized variance decomposition under the frequency band *d* = (*a*, *b*): *a*, *b* ∈ (− *π*, *π*), *a* < *b* as

$$\left( {\tilde{\Theta }_{d} } \right)_{j,k} = {{\left( {\Theta_{d} } \right)_{j,k} } \mathord{\left/ {\vphantom {{\left( {\Theta_{d} } \right)_{j,k} } {\sum\limits_{k} {\left( {\Theta \infty } \right)_{j,k} .} }}} \right. \kern-\nulldelimiterspace} {\sum\limits_{k} {\left( {\Theta \infty } \right)_{j,k} .} }}$$

## Empirical results and discussion

In this section, the results obtained from descriptive statistics, Correlation Analysis, Diebold-Yilmaz (2012) & Barunik and Krehlik (2017) have been provided. The detailed discussion is as below:

### Descriptive statistics

Descriptive statistics of 1668 observations across ten financial markets and two green bonds is presented in Table 2. It is observed that the mean return of all the variables is positive. RBSE and RENA exhibit minimum and maximum daily return, respectively. Standard deviation value of 0.0148 identifies RLSE to be the most volatile among other variables. It is interesting to note that all the variables display left skewness, indicating moderate skewness and existence of an asymmetric tail expanding to negative values. Kurtosis value of all the variables is greater than zero, signifying leptokurtic distributions with more peaks and fat tails. Results of both skewness and kurtosis reject data normality and the same is confirmed by Jarque–Bera test. Augmented Dickey Fuller Test is employed to check the stationarity of constituent series. Each series denotes P value to be less than 5% indicating integration of log returns at I (0). This study uses data extending from 2015 to 2021 which contains COVID period too. Since some observations are found in abnormal period, we employ Chow structural break test to check the structural break points. It is seen that structural breaks are not significant statistically to European financial markets and green bond. Hence, we continue to examine the connectedness without dividing the data in different time intervals.

**Table 2:** Descriptive Statistics

	Minimum	Maximum	Mean	Stdev	Skewness	Kurtosis	JB Test	ADF test	Nobs
RLSE	− 0.09	0.07	0.00	0.01	− 0.40	3.73	0.0***	0.00***	1668
RSSE	− 0.10	0.06	0.00	0.01	− 1.22	12.70	0.00***	0.00***	1668
ROSE	− 0.09	0.05	0.00	0.01	− 0.87	6.97	0.00***	0.00***	1668
RDSME	− 0.07	0.05	0.00	0.01	− 0.48	3.23	0.00***	0.00***	1668
RASE	− 0.14	0.10	0.00	0.01	− 1.22	16.62	0.00***	0.00***	1668
RFFSE	− 0.13	0.10	0.00	0.01	− 0.77	11.52	0.00***	0.00***	1668
RNSDS	− 0.11	0.06	0.00	0.01	− 0.89	8.54	0.00***	0.00***	1668
RBSE	− 0.15	0.07	0.00	0.01	− 1.70	21.02	0.00***	0.00***	1668
RNSDI	− 0.07	0.04	0.00	0.00	− 0.51	5.80	0.00***	0.00***	1668
RENA	− 0.11	0.08	0.00	0.01	− 0.91	11.16	0.04*	0.00***	1668
RBMSCIGB	− 0.03	0.02	0.00	0.00	− 0.63	6.66	0.00***	0.00***	1668
RSPDR	− 0.02	0.02	0.00	0.00	− 0.58	6.39	0.00***	0.00***	1668

This table provides the descriptive statistics of select returns of European financial market and green bond. Stdev indicates the standard deviation, JB Test is Jarque–Bera Test applied to check the normality while ADF Test is Augmented Dickey Fuller test for stationarity. * and *** depict the significance level at 5% and 0.01% level. Table 4: BK (2017) test results of connectedness of constituent series

**Fig. 2:**
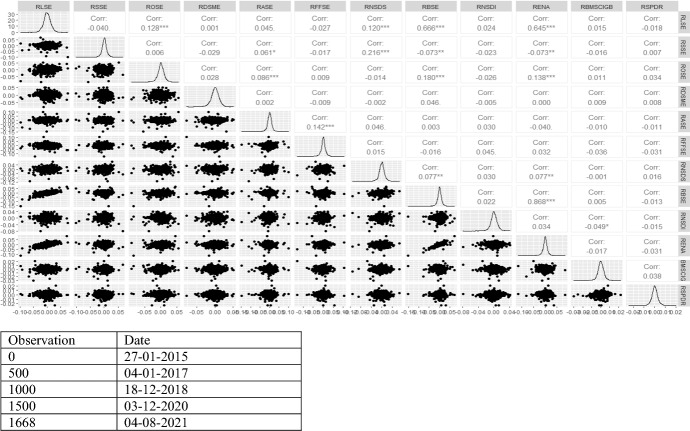
Correlation analysis

Figure 2 shows the overall distribution pattern along with pairwise correlation between green bond and financial markets together. It is confirmed from the figure that no market is normally distributed. RENA and RBSE are witnessed with high positive correlation (0.868) followed by RBSE and RLS (0.666). These financial markets have similar segment, and they are related assets. Hence, it is not surprising that they have the high degree of association. However, green bond, i.e. RBMSCIGB and RSPDR are not backed by strong correlation with other financial markets. It is verified further employing Diebold and Yilmaz (2012) and Barunik et al. (2018) for the total connectedness.

**Fig. 3:**
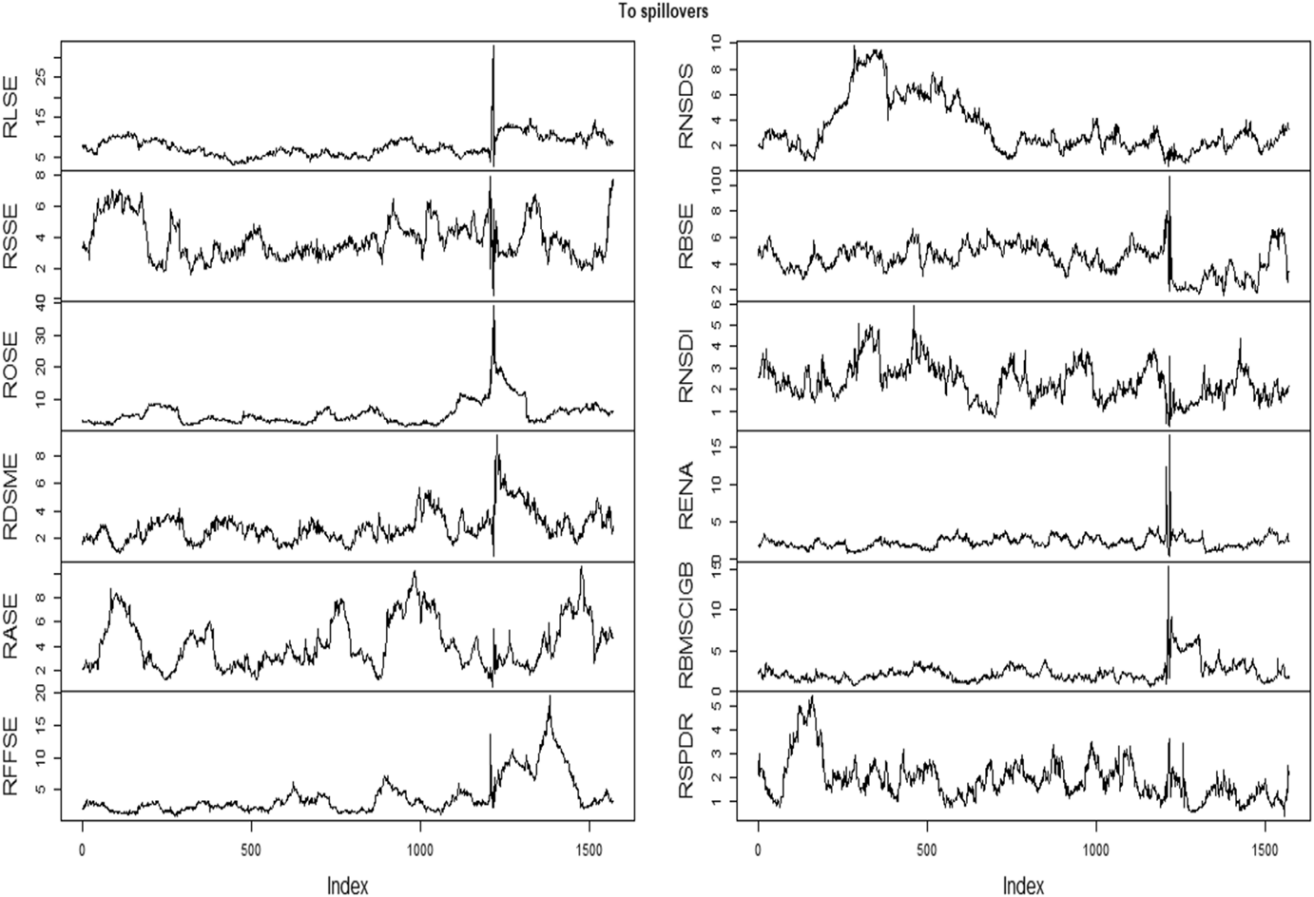
Directional spillover from other constituent markets

**Fig. 4:**
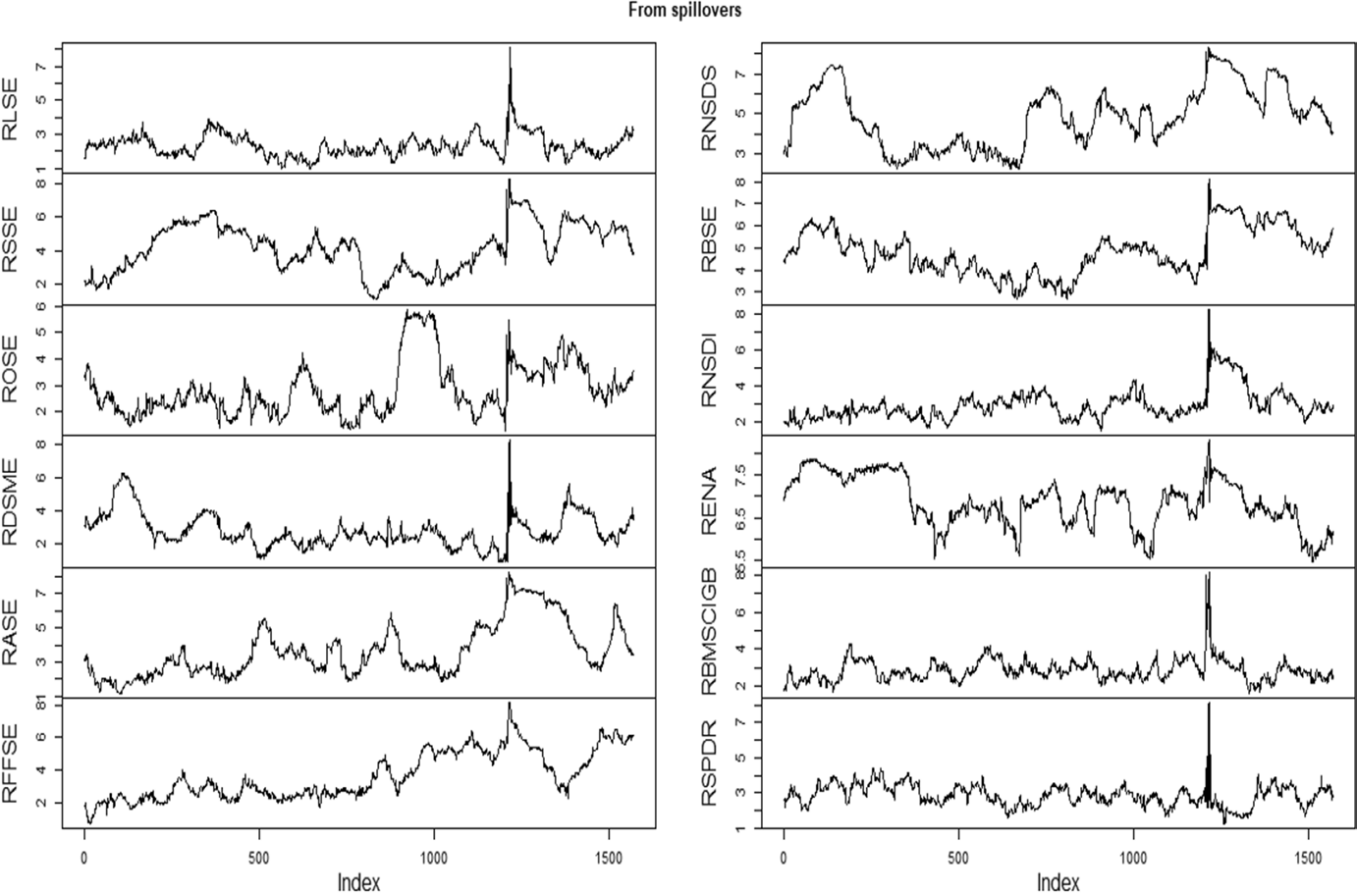
Directional spillover to other constituent markets. Source- Author’s calculation

Table 3 documents the spillover result using Diebold and Yilmaz (2012) model in which within and cross-market spillover is presented by diagonal and off-diagonal element of the matrix. The terminology “From” depicts the spillover derived from other markets, while “To” represents the spillover contributed to constituent markets. It is seen that RBSE obtains highest spillover (4.68) followed by RENA (4.61) from other markets considered under examination, while RSPDR derives the least spillvoer (0.21). Further, RBSE is the highest contributor of the volatility followed by RENA with 5.35 and 5.01%, respectively. Surprisingly, RSPDR and RNSD are least contributors to the other markets with 0.18%. After computing the connectedness between green bond and financial markets using Diebold and Yilmaz (2012), it is displayed in graphical presentation. Figures 3 and 4 present the volatility spill over “from” and “to” other constituent markets, respectively. We notice that higher values of gross directional spill over are found during the beginning of 2020 (pandemic covid outbreak). The same pattern is shown in Fig. 3. Surprisingly, the green bond (RSPDR) has high directional spill over during the end of 2015 but not in covid outbreak. It is found that financial crisis experiences the more dynamic linkages due to which the diversification opportunities cannot be possible. The findings of our study corroborate the Palanska (2018).

**Table 3:** Spillover between European financial market and green bond using Diebold and Yilmaz (2012)

	RLSE	RSSE	ROSE	RDSME	RASE	RFFSE	RNSDS	RBSE	RNSD	RENA	RBMSCIGB	RSPDR	FROM
RLSE	51.76	0.28	1.59	0.08	0.55	0.48	0.36	22.67	0.04	21.39	0.72	0.09	4.02
RSSE	0.31	80.41	1.41	0.46	1.88	4.72	8.32	0.56	0.14	1.13	0.43	0.22	1.63
ROSE	2.73	0.17	87.41	0.19	0.89	0.49	0.54	4.34	0.15	2.69	0.31	0.09	1.05
RDSM	0.09	0.74	0.66	92.92	3.04	0.36	0.73	0.29	0.60	0.07	0.41	0.09	0.59
RASE	0.61	0.52	6.28	0.24	79.27	10.35	0.15	1.00	0.21	0.76	0.40	0.21	1.73
RFFSE	0.68	0.23	4.41	0.05	2.74	89.84	0.12	0.88	0.25	0.41	0.27	0.12	0.85
RNSDS	0.86	11.59	2.71	0.48	6.14	2.59	73.64	0.55	0.04	0.43	0.16	0.80	2.20
RBSE	18.57	0.23	2.14	0.32	0.37	0.19	0.24	43.86	0.10	32.70	1.25	0.02	4.68
RNSDI	0.26	0.59	0.42	0.96	0.21	0.29	0.05	0.05	96.67	0.08	0.20	0.23	0.28
RENA	18.24	0.25	1.32	0.17	0.43	0.06	0.17	33.53	0.05	44.71	1.01	0.05	4.61
RBMSCIGB	0.03	0.36	0.09	0.30	0.16	0.59	0.16	0.14	0.24	0.28	97.36	0.28	0.22
RSPDR	0.16	0.27	0.69	0.01	0.05	0.18	0.26	0.17	0.29	0.13	0.30	97.46	0.21
TO	3.55	1.27	1.81	0.27	1.37	1.69	0.93	5.35	0.18	5.01	0.46	0.18	22.06

This table shows the results obtained from spillover between European financial market and green bond using Diebold Yilmaz (2012). “From” indicates the spillover derived, while “To” represents the spillover contributed to other market

To refine the connectedness between green bond and financial markets, the study further employs BK (2017) test which is shown in Table 4, computing its net pairwise connectedness of twelve series. It furnishes the magnitude and direction of the dynamic linkages or spill over “from” and “to” of the various markets studied in this paper in different frequency cycle. For the analysis purpose, the frequency cycle is categorized into three parts: frequency 1 (1 day to 10 days), frequency 2 (10 days to 15 days) and frequency 3 (15 days to infinity). WTH stands for within which shows the within connectedness across the constituent markets, while ABS stands for absolute connectedness into three different frequencies (Gupta et al. 2020). Further, NET is the difference between TO_ABS-FROM_ABS spill over. In existing literature works, the transmission of market volatility was investigated employing the spillover index, the systematic co-movement and causality analytical framework (Ortas and Moneva, 2013). However, in this paper, we focus on frequency connectedness for empirical importance considering that volatility shocks vary over the time which impact the future uncertainty.

**Table 4:** BK (2017) test results of connectedness of constituent series

Frequency 1 The spillover table for band: 3.14 to 0.79 (1 days to 10 days)													
	RLSE	RSSE	ROSE	RDSME	RASE	RFFSE	RNSDS	RBSE	RNSDI	RENA	RBMSCIGB	RSPDR	FROM_ABS
RLSE	38.63	0.27	0.88	0.07	0.52	0.37	0.34	14.71	0.03	14.07	0.31	0.08	2.64
RSSE	0.15	61.49	1.02	0.3	0.96	3.09	4.75	0.34	0.13	0.88	0.18	0.19	1
ROSE	1.4	0.16	67.66	0.14	0.48	0.23	0.51	2.72	0.09	1.59	0.16	0.08	0.63
RDSME	0.05	0.59	0.55	70.54	1.24	0.28	0.62	0.27	0.36	0.04	0.24	0.09	0.36
RASE	0.35	0.24	4.12	0.22	53.75	4.93	0.14	0.65	0.18	0.51	0.17	0.08	0.96
RFFSE	0.53	0.21	2.51	0.05	1.12	67.16	0.12	0.58	0.23	0.14	0.2	0.1	0.48
RNSDS	0.51	6.35	2.02	0.28	4.42	1.83	58.13	0.27	0.04	0.22	0.13	0.78	1.4
RBSE	13.65	0.21	1.27	0.24	0.31	0.16	0.21	31.4	0.1	23.52	0.64	0.01	3.36
RNSDI	0.11	0.59	0.34	0.71	0.08	0.1	0.03	0.01	72.35	0.07	0.13	0.19	0.2
RENA	13.45	0.22	0.71	0.16	0.29	0.05	0.17	23.93	0.04	32.76	0.62	0.05	3.31
RBMSCIGB	0.02	0.28	0.07	0.2	0.13	0.41	0.13	0.13	0.22	0.26	67.58	0.27	0.18
RSPDR	0.16	0.16	0.49	0	0.05	0.11	0.23	0.15	0.17	0.13	0.29	66.34	0.16
TO_ABS	2.53	0.77	1.16	0.2	0.8	0.96	0.6	3.65	0.13	3.45	0.26	0.16	14.68
TO_WTH	3.52	1.07	1.62	0.27	1.11	1.34	0.84	5.07	0.18	4.79	0.35	0.22	
NET	− 0.11	− 0.23	0.53	− 0.16	− 0.16	0.48	− 0.80	0.29	− 0.07	0.14	0.08	0	

Frequency 2 The spillover table for band 0.79 to 0.39 (10–15 days)													
	RLSE	RSSE	ROSE	RDSME	RASE	RFFSE	RNSDS	RBSE	RNSDI	RENA	RBMSCIGB	RSPDR	FROM_ABS
RLSE	8.12	0.01	0.38	0.00	0.03	0.07	0.02	4.73	0.00	4.38	0.22	0.00	0.82
RSSE	0.09	12.08	0.28	0.10	0.50	0.97	2.29	0.14	0.00	0.17	0.18	0.02	0.40
ROSE	0.74	0.00	12.26	0.03	0.24	0.14	0.02	0.86	0.04	0.58	0.08	0.01	0.23
RDSME	0.03	0.12	0.09	14.37	1.02	0.05	0.08	0.01	0.15	0.03	0.10	0.00	0.14
RASE	0.16	0.17	1.16	0.01	15.18	2.99	0.01	0.24	0.03	0.18	0.18	0.08	0.43
RFFSE	0.10	0.02	1.06	0.00	0.89	13.72	0.00	0.17	0.01	0.13	0.06	0.01	0.20
RNSDS	0.14	3.25	0.43	0.13	1.05	0.50	10.04	0.12	0.00	0.09	0.03	0.01	0.48
RBSE	3.04	0.01	0.50	0.05	0.05	0.02	0.02	7.75	0.00	5.71	0.37	0.01	0.81
RNSDI	0.08	0.01	0.05	0.16	0.06	0.09	0.01	0.01	15.48	0.00	0.06	0.03	0.05
RENA	2.95	0.02	0.36	0.01	0.09	0.01	0.01	5.92	0.00	7.46	0.25	0.00	0.80
RBMSCIGB	0.01	0.06	0.02	0.06	0.03	0.11	0.02	0.00	0.02	0.01	18.41	0.01	0.03
RSPDR	0.01	0.07	0.12	0.01	0.01	0.03	0.03	0.01	0.07	0.01	0.01	19.04	0.03
TO_ABS	0.61	0.31	0.37	0.05	0.33	0.41	0.21	1.02	0.03	0.94	0.13	0.01	4.42
TO_WTH	3.55	1.80	2.14	0.27	1.91	2.41	1.21	5.91	0.16	5.46	0.74	0.09	
NET	− 0.21	− 0.09	0.14	0.13	0.10	0.21	− 0.27	0.21	− 0.02	0.14	0.10	− 0.02	

Frequency 3 The spillover table for band 0.39 to 0.20 (15 days to infinity)													
	RLSE	RSSE	ROSE	RDSME	RASE	RFFSE	RNSDS	RBSE	RNSD	RENA	RBMSCIGB	RSPDR	FROM_ABS
RLSE	5.00	0.00	0.33	0.00	0.00	0.04	0.00	3.23	0.00	2.94	0.18	0.00	0.56
RSSE	0.08	6.84	0.11	0.06	0.43	0.66	1.28	0.08	0.00	0.08	0.07	0.00	0.24
ROSE	0.58	0.00	7.50	0.02	0.17	0.12	0.01	0.75	0.02	0.51	0.07	0.00	0.19
RDSME	0.00	0.03	0.02	8.01	0.77	0.04	0.03	0.01	0.08	0.00	0.06	0.00	0.09
RASE	0.10	0.11	1.00	0.00	10.33	2.43	0.00	0.12	0.01	0.08	0.05	0.06	0.33
RFFSE	0.06	0.00	0.83	0.00	0.74	8.95	0.00	0.14	0.00	0.14	0.01	0.00	0.16
RNSDS	0.21	2.00	0.27	0.08	0.67	0.26	5.46	0.16	0.00	0.12	0.01	0.01	0.32
RBSE	1.87	0.01	0.38	0.03	0.01	0.01	0.01	4.72	0.00	3.47	0.25	0.00	0.50
RNSDI	0.07	0.00	0.03	0.09	0.07	0.09	0.01	0.02	8.84	0.00	0.01	0.02	0.03
RENA	1.84	0.01	0.25	0.00	0.04	0.01	0.00	3.68	0.00	4.49	0.15	0.00	0.50
RBMSCIGB	0.00	0.03	0.00	0.04	0.00	0.07	0.00	0.01	0.00	0.01	11.37	0.00	0.01
RSPDR	0.00	0.05	0.08	0.01	0.00	0.04	0.00	0.00	0.05	0.00	0.00	12.08	0.02
TO_ABS	0.40	0.19	0.28	0.03	0.24	0.31	0.11	0.68	0.01	0.61	0.07	0.01	2.95
TO_WTH	3.73	1.74	2.56	0.26	2.26	2.91	1.05	6.35	0.14	5.70	0.67	0.08	
**NET**	− **0.16**	− **0.05**	**0.09**	− **0.06**	− **0.09**	**0.15**	− **0.21**	**0.18**	− **0.02**	**0.11**	**0.06**	− **0.01**	

This table furnishes the results of BK (2017) test for the connectedness in form of three different time horizons. Frequency 1, 2 and 3 shows the spillover in short, medium and long period among the considered markets under examination.

Frequency 3 The spillover table for band 0.39 to 0.20 (15 days to infinity)

Bold value indicates the net Spillover among examined variable

The volatility contribution of green bond and selected financial markets is shown in Table 4, Brussels stock exchange (RBSE) derives highest volatility (3.36) followed by Euronext Amsterdam (RENA) (3.31). Surprisingly, the same two stock markets are the highest contributors to the volatility at 3.65 and 3.45, respectively.

It indicates that RBSE and RENA add high risk to the portfolio compared to rest of the financial markets and green bond. Further, in medium period (frequency 2), RLSE and RBSE are two major stock markets which derive high volatility at 0.82 and 0.81, respectively. RBSE (1.02) and RENA (0.94) are the major contributors to the volatility in portfolio. In long run, RLSE (0.56) derives the highest volatility followed by RBSE and RENA (0.50). Further, RBSE (0.68) contributes the highest volatility followed by RENA (0.61). It is observed that RBSE and RENA both are contributors to the volatility in all frequency cycles (frequency 1 to frequency 3). Therefore, an investor should avoid these two stocks in their portfolio.

Finally, the net directional connectedness for each specific market is computed. If any market has positive net spill over, it can be called spill over contributor and negative net spill over indicates the net receiver which receives spill over from other markets (TN-Lan et al. 2021). The spill over contributors and receivers differ from frequency to frequency. On this note, we find that the net contributors to the volatility are ROSE, RFFSE, RBSE, RENA, RBMSCIGB to RLSE, RBSE, RDSME, RASE, RNSDS and RNSDI in short run. Surprisingly, RSPDR is neither net contributor nor net receiver. It signifies that holding green bond (RSPDR) in portfolio will mitigate the risk among financial markets. In the medium run, ROSE, RDSME, RASE, RFFSE, RBSE, RENA and RBMSCIGB are net contributors to RLSE, RSSE, RNSDS, RNSDI and RSPDR. Further, ROSE, RFFSE, RBSE, RENA and RBMSCIGB are net contributors and RLSE, RSSE, RDSME, RASE, RNSDS, RNSD and RSPDR are net receiver. In medium and long period (frequency 2 and frequency 3), it is evident that similar market segments in both green bond and financial markets are both net contributors and receivers (Yao et al. 2018). From the above results of frequency domain, it is also noticed that the total connectedness of these twelve indices is greater in short run (14.68) than medium run (4.42) and long run (2.95) which provides few opportunities in short run for the portfolio diversification.

## Conclusions and policy implications

To elaborate upon the growing significance of green bonds in investor portfolio, assessment of co-movement among green bonds and stock market movement has emerged as a prominent research area. Such studies gain relevance at times of crises. This study provides fresh evidence of co-movement between green bonds and OECD financial markets using Diebold and Yilmaz and Barunik tests. Since limited studies have explored the co-movement between green bonds and European financial markets, this study gains prominence.

This study employs Diebold and Yilmaz (2012) and Barunik & Krehlic (2017) test to examine the connectedness between green bonds and OECD countries of European Financial Markets. Results from short run, medium term and long run, from both the models, identify volatility across all frequency cycles (frequency 1 to frequency 3). Hence, RBSE and RENA are identified as high-risk markets in comparison with the other OECD financial markets and green bonds. Owing to their maximum volatility contribution, stocks from these markets should be avoided in an investor’s portfolio. Results from Barunik & Krehlic (2017) tests identify S&P Dow Jones Green Bond Index (RSPDR) as neutral volatility contributor and receiver, suggestive of its potential to mitigate risk, if included in portfolio in the short run. Results from medium and long period (frequency 2 and frequency 3) models identify similar market segments to be volatile. Results from various frequency domains establish that total connectedness of all the twelve indices is maximum in the short run (14.68), in comparison with medium run (4.42) and long run (2.95), respectively, implying fewer opportunities in short run for portfolio diversification. It has been observed that climate mitigation and adaptation effects only materialize in the long term, while many investors have a very short time horizon for investments. Often, long-term climatic trends are not figured into their investment calculation. The recommended time-horizon for implementing this measure should be short-term as this measure will be the necessary first step towards a common framework for determining which projects should be financed by green bonds.

The paper’s findings provide useful implications for investors, portfolio managers, financial managers, and particularly policy makers. Firstly, the study recommends that investments in Brussels stock exchange (RBSE) and Euronext Amsterdam (RENA) should be avoided, since they are identified as potential volatility contributors across all frequency cycles. Secondly, neutral contribution by S&P Dow Jones Green Bond Index (RSPDR) suggests its inclusion in investor’s portfolio as a risk inhibitor. Thirdly, results from Barunik & Krehlic (2017) provide similar results across medium- and long-term frequency cycles, implying elevated volatility in the longer run. This study thus advocates that for effective risk transmission, investors in OECD economies should hold green bonds for comparatively shorter time periods only.

An increase in green bond investments would increase the sustainable market capital, which, when deployed, serves to benefit the society at large. The main motive behind green bond investments should be to invest for a sustainable environment followed by the possibility to gain a combined financial and environmental return. In addition to the financial attributes, investors find a utility function in the green bonds that accounts for the premium price that these investors should except. However, a further investigation into the cost of issue and the returns can be clearly identified as further scope of this study.

Our findings are subject to limitations that provides scope for further research. Future studies can examine spill over impact between green bonds and OECD financial markets using Copula and BEKK model. As this study is limited to examining the volatility correlation between green bonds and stock markets, it does not measure out-of-sample forecasting. To examine the dynamic relationship between each paired market, the time-varying copula model can be adopted. Thus, this study can be extended using different stochastic volatility (SV) models to measure both in-sample and out-of-sample forecasting. SV model has gained much attention in financial market for its contribution to option pricing theory. SV model which is in contrast of the Garch Model has underlying ideas of SV type models in which the volatility itself is assumed to follow a stochastic process. Therefore, extension of this work in SV model will enhance the function. Further, this research model can also be explored using machine learning and deep learning forecasting models of volatility.
